# Synthesis of Large Area Graphene for High Performance in Flexible Optoelectronic Devices

**DOI:** 10.1038/srep16744

**Published:** 2015-11-18

**Authors:** Emre O. Polat, Osman Balci, Nurbek Kakenov, Hasan Burkay Uzlu, Coskun Kocabas, Ravinder Dahiya

**Affiliations:** 1 Electronics and Nanoscale Engineering, University of Glasgow, Glasgow, G12 8QQ, UK; 2 Department of Physics, Bilkent University, 06800, Ankara, Turkey

## Abstract

This work demonstrates an attractive low-cost route to obtain large area and high-quality graphene films by using the ultra-smooth copper foils which are typically used as the negative electrodes in lithium-ion batteries. We first compared the electronic transport properties of our new graphene film with the one synthesized by using commonly used standard copper foils in chemical vapor deposition (CVD). We observed a stark improvement in the electrical performance of the transistors realized on our graphene films. To study the optical properties on large area, we transferred CVD based graphene to transparent flexible substrates using hot lamination method and performed large area optical scanning. We demonstrate the promise of our high quality graphene films for large areas with ~400 cm^2^ flexible optical modulators. We obtained a profound light modulation over a broad spectrum by using the fabricated large area transparent graphene supercapacitors and we compared the performance of our devices with the one based on graphene from standard copper. We propose that the copper foils used in the lithium-ion batteries could be used to obtain high-quality graphene at much lower-cost, with the improved performance of electrical transport and optical properties in the devices made from them.

Carbon based nanomaterials have provided a new perspective in electronics owing to their very high charge carrier mobility and nanoscale dimensions. In particular, the carbon nanotubes^1^ and graphene^2^ have been shown to have promising performances suitable for high frequency electronics, which will be needed in emerging applications such as smart cities and mobile health. It is essential to have high quality material for high-performance and scaling up of electronics on large areas. In this regard, the wafer scale synthesis of aligned arrays of single-walled carbon nanotubes on quartz^3^ and sapphire crystals^4^ are worth noting. As a two-dimensional crystal of carbon atoms, graphene is a basic building block of many carbon allotropes such as zero dimensional fullerene, one dimensional carbon nanotube, and three dimensional graphite^5^. Since first observation of strong ambipolar electric field effect in the graphitic films^6^ by Andre Geim and Kostya Novoselov, there has been a tremendous effort from scientists to explore and enhance the electronic properties of graphene. These include ground-breaking discoveries such as experimental observation of the half integer quantum hall effect^7^,^8^, constant minimum conductivity in vicinity of charge carriers^9^, ballistic transport at room temperature^5^ and Klein paradox^10^. Graphene is also a viable material for optoelectronics because of its broad optical response and gate-tunable optical properties^11^. One can control the absorption of graphene via electrostatic doping^12^. The current focus of graphene research is to obtain high quality graphene over large areas to realize electronic and optic applications with advanced mechanical functionalities such as flexible transparent electrodes^13^ or large area touch sensor devices^14^.

Graphene layers can be isolated chemically from bulk graphite with intercalation of atoms or molecules through the graphite^15^, yielding the separation of graphene layers. However, partial separation of the bulk graphite results in a dispersion that contains variety of graphene fragments and graphitic films^16^. To overcome this non-uniformity of the resulting graphene layers, chemical reduction of graphene oxide^17^ and chemically functionalized graphene^18^ were introduced. As a common drawback, these materials suffer from low conductivity, therefore, it is challenging to achieve large area graphene from them with state of the art transport properties. A promising scalable route is the chemical vapour deposition (CVD) of graphene on copper (Cu) foils^19^. Cu is preferred in CVD of graphene because of low solubility limit of carbon in Cu, which makes graphene growth procedure self-limited^19^. This property of Cu provides a straightforward method to synthesize single layer graphene. The large area synthesis of graphene by using Cu in CVD has been investigated since 2009^20^,^21^ and many breakthrough improvements on the quality of graphene films have been reported since then^13^,^19^,^22^.

The surface quality of the Cu foils and the transfer process greatly influence the performance and reliability of graphene based devices. Previous works have showed that the morphology of the Cu surface is one of the key parameters to have better quality graphene films^23^. To achieve the continuous and high quality graphene film over large area; impurities, defects, grain boundaries and other surface features that can serve as a nucleation seed should be carefully engineered^23^. To that end, variety of techniques to smoothen the Cu surfaces have been reported. For example, Luo *et al.*^24^ used standard electro-polishing technique to smoothen the Cu surfaces. Their Raman investigation implied better quality graphene with higher surface coverage compared to the unpolished Cu samples^24^. Similar electrochemical polishing technique was used in the work by Yan. *et al.*^25^. Together with the high pressure annealing, they produced hexagonal single crystal graphene domains nearly 2 mm in size^25^. Alternatively, melted Cu surfaces were also used to form single crystal hexagonal graphene flakes in previous works^26^,^27^. It has been known that the quality of the Cu surface directly effects the charge carrier transport properties of the resulting graphene layer^28^. Orofeo *et al.*^28^ demonstrated the hole mobility of the graphene grown on the hetero-epitaxial Cu film is nearly 10 times greater than that of the graphene grown on the standard Cu foil by chemical vapor deposition. Above technologies including Cu polishing and deposition techniques add extra cost and time consuming chemical process steps which are not effective in the case of large area synthesis of graphene.

In this paper, we demonstrate that the commercially available ultra-smooth Cu foils (which are generally used in Lithium-ion batteries) can help to open new avenues for large area device applications with the improved quality of the graphene films. We studied the synthesis of graphene on commercially available smooth Cu foils and we provided a detailed comparison between the graphene synthesized on ultra-smooth Cu foils and the one synthesized on the standard Cu foils in terms of structural, electrical and optical properties. We observed a stark improvement in the electrical performance of the transistors realized on our graphene films. Then, to demonstrate the advantage of high quality graphene films from ultra-smooth Cu foils, we fabricated 400 cm^2^ flexible graphene electrodes and enlarged 160 times the previously reported (2.5 cm^2^) supercapacitor structure^29^. To show the promises of our graphene films in electro-optic devices, we tested and compared the effects of graphene quality in large area supercapacitors. We anticipate that usage of commercially available ultra-smooth Cu foils for large area graphene growth would lead to higher performance devices especially for optical and electronic applications where low device variations are desired.

## Results

**Figure 1:**
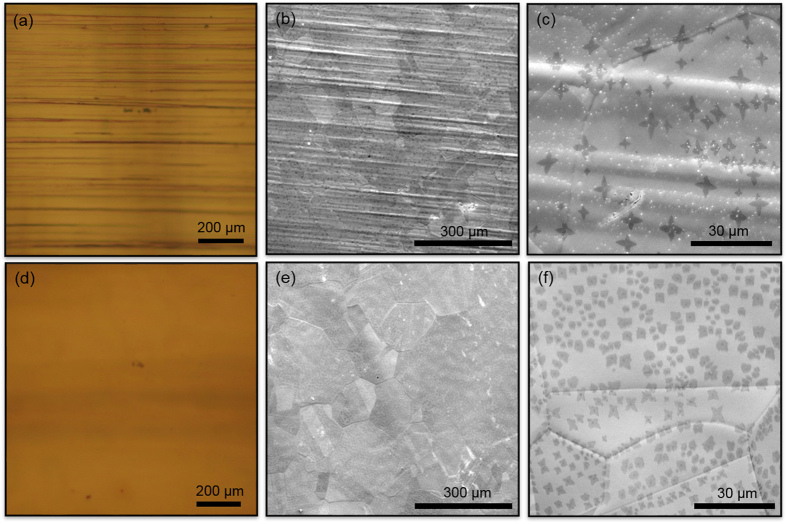
Effects of Cu surface morphology on graphene growth: (**a**) The optical microscope image of rough Cu foil (*Alfa Aesar* Cu foil) commonly used in graphene growth. Deep scratches on the rough Cu foil due to the rolling process are clearly visible (**b**) Scanning electron microscopy (SEM) image of the rough Cu surface. (**c**) The magnified SEM image of graphene flakes on rough Cu foil surface. The growth was terminated after 10 seconds to obtain dispersed graphene flakes. (**d**) Optical microscope image of commercially available ultra-smooth Cu foil (Mitsui mining and smelting co. LTD., B1-SBS) and (**e**) SEM image of ultra-smooth Cu surface. (**f**) Ultra-smooth Cu surfaces with graphene flakes. The density and shape of the graphene flakes are different on the rough and smooth foils. The sizes of individual grains along the Cu surface are clearly visible.

We start with the demonstration of the high quality single layer graphene synthesis on commercially available ultra-smooth Cu foils. In order to compare the surface quality, we used relatively rough *Alfa Aesar* Cu foil which is commonly used for CVD of graphene (Fig. 1(a–c)) and commercially available ultra-smooth surface Cu foils (*Mitsui mining and smelting co., LTD, B1-SBS*) (Fig. 1(d–f)) in the same CVD chamber. Deep trenches on the surface of rough copper foils are clearly seen in both optical microscope (Fig. 1(a)) and scanning electron microscope images. (Fig. 1(b,c)). On the other hand, commercially available smooth copper foils show uniform surface properties (Fig. 1(d–f)). We investigated the surface topography of the ultra-smooth Cu foils via scanning electron microscopy (SEM) and atomic force microscopy (AFM). The root mean square (RMS) surface roughness of the ultra-smooth Cu is around 100 nm before annealing which is two times lower than the value reported for the Cu foils commonly used in graphene growth^30^.

**Figure 2:**
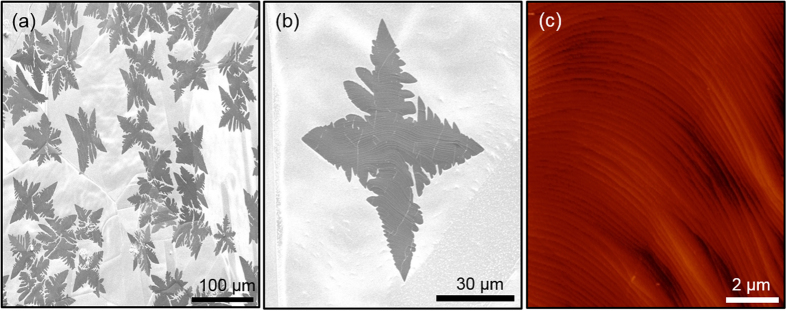
Graphene flake formation at early stages of growth: (**a,b**) SEM images of the surface of the ultra-smooth Cu partially covered by graphene flakes. To achieve ~1100 μm^2^ flakes, methane gas was flushed into chamber for 5 seconds then left for cooling to room temperature. Smoothness of the surface and size of the grain boundaries of the Cu are clearly visible. (**c**) AFM image showing the surface topography of full graphene layer on the ultra-smooth Cu foil. Buckling on the graphene surface is clearly visible.

By sending the methane gas into the chamber with different intervals, we obtain both the graphene flakes (Fig. 2(a,b)) and full coverage graphene film (Fig. 2(c)) on the ultra-smooth Cu foils. Figure 2(a) shows the SEM image of Cu surface partially covered by graphene flakes. The average size of the flakes are ~1100 μm^2^ in the form of a snow-flake (Fig. 2(b)). Also we observed that the flake size and shape are directly related to the ratio of flow rate and partial pressure of hydrogen to methane gases, as reported in previous work^31^.

As a next step, the partial pressure and flow of the gases were set to have full coverage single layer graphene on both the ultra-smooth Cu foils and rough copper foils. We followed the similar growth conditions as in ref. 22. According to our observations, the formation of continuous graphene layer is initiated with single crystal graphene flakes. Increasing the growth time, under the same methane flow rate and partial pressure, results in the formation of continuous graphene layer. Moreover, the synthesized graphene flakes and continuous graphene layers includes ripples (Fig. 2(c)) because of the physical instability of perfectly flat graphene layer^32^,^33^,^34^,^35^.

To investigate the effect of the surface roughness on the quality of the graphene layer, both the ultra-smooth Cu and the rough Cu foils were placed in the same growth chamber so as to expose them to the same growth conditions. Once the graphene layers were formed on the both the smooth and the rough Cu surfaces, we transferred them to 100-nm-thick SiO_2_ coated Si wafers for structural characterization and transistor applications. (Supplementary Fig. S1)

**Figure 3:**
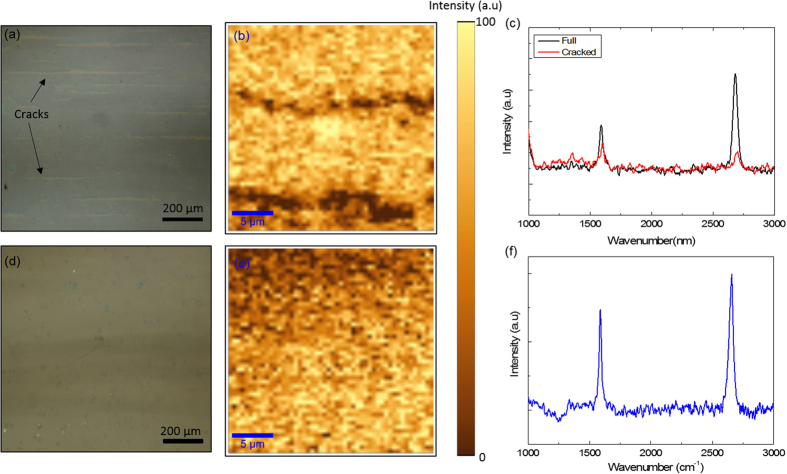
Raman investigation of graphene synthesized on rough and smooth copper foils: (**a**) The optical microscope image of rough-Cu-graphene on SiO2/Si. Formation of the cracks on the graphene layer is a due to the deep trenches of rough-surface Cu foil. (**b**) Raman mapping of rough-Cu-graphene transferred on SiO2/Si. Cracked and graphene free areas appear as low intensity dark areas in Raman mapping image. (**c**) Raman spectrum of rough-Cu-graphene taken from fully graphene coated and graphene-free cracked areas. The G and 2D bands are hardly detectable in the cracked areas and the Raman intensity is very low when compared to the Raman signal taken from full coverage graphene area. (**d**) Optical microscope image of smooth-Cu-graphene transferred on SiO2/Si. In contrast to rough-Cu-graphene there is no graphene-free areas throughout the 1 cm^2^ area. (**e**) Raman mapping of smooth-Cu-graphene. Due to high surface coverage of the graphene layer we didn’t observe any low intensity graphene-free areas in Raman mapping. (**f**) Raman spectrum of smooth-Cu-graphene. We obtain a uniform Raman signal from the smooth-Cu-graphene throughout the 25 μm × 25 μm scan area.

From now on, we will denote the graphene synthesized on commercially available ultra-smooth Cu foils as “smooth-Cu-graphene” and the graphene synthesized on standard rough copper foils as “rough-Cu-graphene”. To present a structural comparison, we performed Raman mapping for both the smooth-Cu-graphene and rough-Cu-graphene. Figure 3(a) shows the optical microscope image of rough-Cu-graphene transferred on SiO_2_/Si. Rough-Cu-graphene includes longitudinal cracks along the graphene layer. These cracks are the graphene-free areas and resulted from the deep trenches of the rough Cu surface. Graphene synthesized on these trenches are not compatible to transfer techniques since they are formed in different height with respect to overall surface and when transferred to solid substrates these areas remain without graphene. Figure 3(b) shows the Raman mapping of the rough-Cu-graphene. Longitudinal cracks on the surface appears as low intensity black areas. Figure 3(c) shows the comparison of Raman spectrum taken from the different parts of rough-Cu-graphene including both the cracks and the full coverage graphene. Due to lacking of graphene in the cracked areas, Raman fingerprints of graphene such as G band and 2D band can hardly be detected in these areas and the Raman intensity is relatively low when compared to full coverage graphene areas. Figure 3(d) shows the optical microscope image of the smooth-Cu-graphene. After transferring to dielectric surfaces we didn’t observe any cracks on the smooth-Cu-graphene. We performed the Raman mapping in same conditions for smooth-Cu-graphene (Fig. 3(e)) and we obtain a stable Raman signal throughout the whole scanning area (25 μm x 25 μm) due to high surface coverage of the smooth-Cu-graphene. Figure 3(f) shows the Raman signal taken from the smooth-Cu-graphene surface. The Raman fingerprints of the single layer graphene, namely the peaks of G band and 2D band are clearly seen in the spectrum. We observed the G peak at 1587 nm with the full width at half maximum (FWHM) of ~23. The FWHM of 2D peak at 2658 cm^−1^ is ~40 and G/2D ratio is ~1.4.

**Figure 4:**
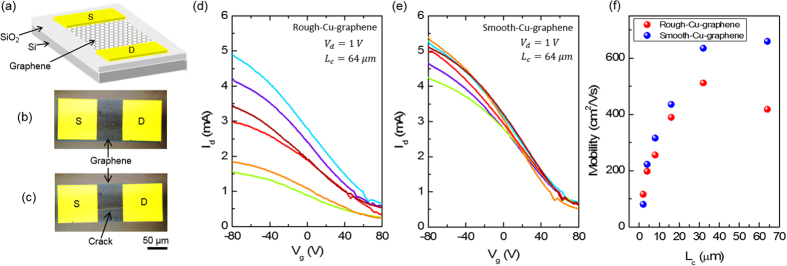
Transport properties of fabricated graphene based transistors: (**a**) Schematic drawing of the fabricated graphene field effect transistor. The transistors use 100 nm thick SiO_2_ as a dielectric and highly doped Si as a back gate electrode. The source (S) and drain (D) electrodes are 50 nm thick thermally evaporated gold. (**b,c**) Optical microscope images of the fabricated transistors based smooth-Cu-graphene and rough-Cu-graphene respectively. The deep scratches on the Cu foils results in visible cracks in transferred graphene. (**d,e**) Transfer curves of the six different transistors with a channel length (L_c_) of 64 μm, based on the smooth-Cu-graphene and rough-Cu-graphene respectively. Drain voltage (V_d_) was kept constant at 1 V during the measurement. The transistors based on rough-Cu-graphene, show significantly larger variation in the on-current values. (**f**) The channel length (L_c_) scaling of the field effect mobility of the transistors. The scattered plot shows the averaged value of the field effect mobility of 10 identical transistors based on the smooth-Cu-graphene and rough-Cu-graphene.

To compare the electronic properties, we fabricated the graphene field effect transistors based on smooth and rough-Cu-graphene. Schematic drawing of the fabricated back-gated transistors are given in Fig. 4(a). Optical microscope images of the devices using smooth and rough-Cu-graphene are given in Fig. 4(b,c) respectively. The graphene-free spots can also cause variations in charge carrier transport and optical properties. This means the sensors in an array (e.g. active matrix display) or electronic devices in a circuit over large area graphene are likely to have non-uniform response. The transfer characteristics of the fabricated graphene transistors using rough and smooth-Cu-graphene are given in Fig. 4(d) and (e) respectively. We recorded the drain current as a function of gate voltage for six different devices at constant drain voltage. The variation of on-current (Fig. 4(d)) is significantly larger for the FETs using rough-Cu-graphene due to the non-uniform graphene formed on rough Cu foils. The calculated field effect mobility of the devices (Fig. 4(f)) scales with the channel length (See Supplementary Fig. S2 for device statistics). As it seems from the scattered plot, for longer channel lengths where the graphene uniformity starts to play critical role on the transport through the channel, field effect mobility values of the devices using smooth-Cu-graphene are enhanced compared to rough-Cu-graphene. This electrical transport measurements over large graphene areas simply shows the effects of usage of smooth Cu foils in CVD synthesis.

**Figure 5:**
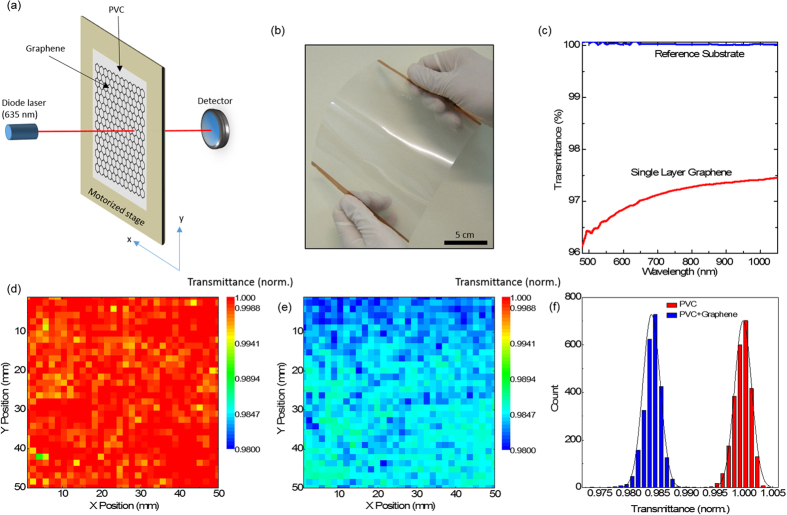
Optical characterization for the flexible graphene electrodes. (**a**) The schematic illustration of the experimental setup. Graphene holding PVC substrates are placed on a motorized stage for the large area optical scan in x and y directions. We used 635 nm diode laser as a light source and recorded the signal changes in the photodetector. (**b**) Photograph of the graphene transferred on PVC substrates by using hot lamination method. During the Cu etching process, we protected the Cu lines at the edges of the sample as an electrical contact pads. (**c**) Transmittance spectra of single layer graphene on PVC substrate. As a reference measurement we used the laminated PVC substrate without graphene. The spot size in the measurement is 2 mm. (**d**) Large area optical scan for a PVC substrate without graphene. We first scanned the reference substrate in x and y directions for 25 cm^2^ area and mapped the normalized transmittance at 635 nm. Due to scattering on the plain PVC surface there are small deviations in transmittance up to ± 0.5%. (**e**) Large area optical scan for a graphene holding PVC substrate. After we transferred smooth-Cu-graphene on PVC, we performed the same large area optical scan and recorded the normalized transmittance and compared with the reference substrate. The transmittance of the single layer graphene is around 1.6% and the total deviation on the 25 cm^2^ graphene area is recorded as ± 0.5% likely due to the scattering from the PVC surface. (**f**) Histogram plot for the large area optical scan of PVC with and without graphene. While the average transmittance of the plain PVC is around 1, the same value for PVC with smooth-cu-graphene is 0.984 due to the small optical absorption single layer graphene defined by the fundamental constants.

After the electrical measurements, to examine the optical properties of smooth-Cu-graphene we performed a large area optical scan. Figure 5(a) shows the schematic illustration for the experimental setup. We used 635 nm diode laser as a light source and with the help of motorized stage we recorded the transmittance on 5 cm x 5 cm graphene area. Figure 5(b) shows the large area graphene (~400 cm^2^) on flexible, 125 μm thick PVC substrate. After the CVD synthesis on ultra-smooth Cu foils, we transferred the graphene to the flexible PVC substrates by using hot lamination method. During the Cu etching, we protected the Cu foils at the edges of the sample to use them as contact electrodes for resistivity measurements and application of bias voltage. The measured total resistance of graphene is superposition of contact resistance and the sheet resistance terms (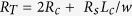). By using transfer length method (TLM) we extracted the sheet resistance of smooth-Cu-graphene as ~2.68 kΩ and contact resistance value as ~0.4 kΩ (Supplementary Fig. S3). Figure 5(c) shows the transmittance measurement of the smooth-Cu-graphene on glass substrates by using conventional spectrometer. Theoretically, free-standing single layer graphene absorbs 2.3% of the incident light. We observed the transmittance of smooth-Cu-graphene on glass substrate is changing from 96% to 97.5% going from UV to near-infrared wavelengths. This is due to the substrate effect during transmittance measurement throughout the broad wavelength range. To check the optical uniformity in large area graphene holding PVC substrate we used the experimental setup given in Fig. 5(a) and mapped the transmittance behaviour in x and y directions. Figure 5(d) shows the transmittance mapping of graphene-free PVC substrate at 635 nm. Due to the scattering on the PVC surface, we observe a small deviation (0.5%) from a fully transparent state. Then we scanned the transmittance of smooth-Cu-graphene transferred on PVC. Figure 5(e) shows the transmittance mapping of smooth-Cu-graphene on PVC. We recorded the absorption of graphene which is stable throughout the scan area and around 1.6%. The difference between the theoretical value (2.3%) for free standing graphene and our result is likely due to the effect of PVC substrate to the measurement. Since the graphene layer is lying on the PVC surface, we also observed the same small deviation resulting from the scattering on the PVC surface in the large area optical scan for graphene. Figure 5(f) shows the histogram for the transmittance mapping of graphene-free PVC and smooth-Cu-graphene on PVC. The transmittance difference due to small optical absorption of graphene and the deviations regarding the PVC surface can be easily seen.

**Figure 6:**
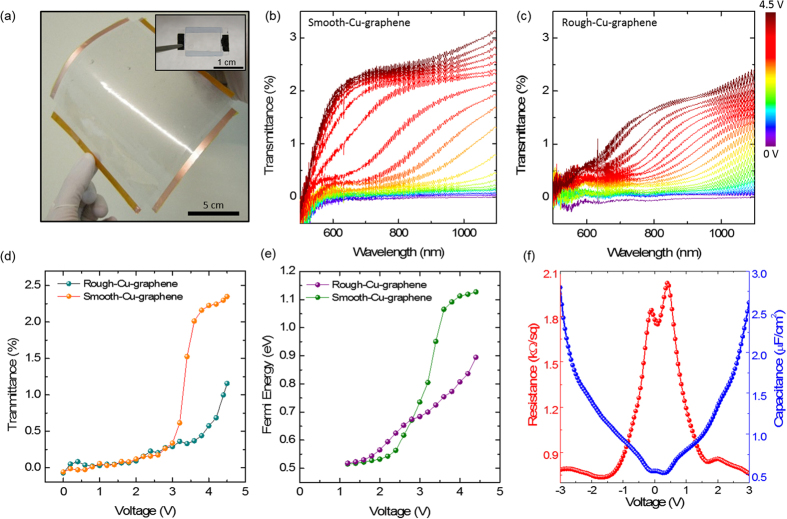
Electro-optic response of large area flexible graphene supercapacitors using rough and smooth-Cu-graphene. (**a**) Photograph of ~400 cm^2^ flexible graphene supercapacitor. Inset shows the previously reported rigid supercapacitor having ~2.5 cm^2^ graphene area. (**b,c**) Transmittance modulation of smooth-Cu-graphene and rough-Cu-graphene based supercapacitors respectively. Increasing the bias voltage shifts the Fermi level of graphene and blocks the optical absorption mechanism of the material. Smooth-Cu-graphene provides more profound light modulation especially in the higher voltage. (**d**) Transmittance versus voltage plot at 700 nm for supercapacitors using both rough and smooth-Cu-graphene. Supercapacitor using rough-Cu-graphene shows less efficient modulation when compared to the one using smooth-Cu-graphene (**e**) Extracted Fermi energy levels with respect to applied bias voltages. Due to lower sheet resistance and uniformity of smooth-Cu-graphene, it gives more efficient Fermi level shift with the applied bias voltage. (**f**) Resistance and capacitance modulation with respect to applied bias voltage. Electrostatic doping of graphene changes the resistance and quantum capacitance of top and bottom graphene electrodes by increasing charge carrier concentration.

Previously we showed that the supercapacitor device structure can provide an efficient electrostatic doping of graphene which cannot be achieved by graphene based transistors using solid insulating dielectric layers^29^. To test and compare the effects of rough and smooth-Cu-graphene in large area electro-optic devices, we fabricated 20 cm × 20 cm graphene supercapacitors by using large area flexible graphene electrodes. Figure 6(a) shows the photograph of large area graphene based flexible supercapacitor. We formed the supercapacitor structure by putting the top and bottom graphene holding flexible PVC electrodes together and filling the gap with ionic liquid electrolyte (1-Butyl-3-methylimidazolium hexafluorophosphate). The inset shows the previously reported rigid device having ~2.5 cm^2^ graphene area. Before forming the supercapacitor structure, ionic liquid electrolyte was soaked into optical cleaning tissue to prevent the electrical shortage between top and bottom graphene electrodes which is due to the sagging of the large area flexible substrate. Figure 6(b,c) show the transmittance modulation of smooth and rough-Cu-graphene based supercapacitors which are operating as voltage controlled optical modulators. To avoid the scattering and absorption effects of PVC we used the unbiased device as reference, then recorded the transmittance changings with the applied bias voltage. Increasing the bias voltage causes the ions inside the electrolyte to form a double layer in the graphene electrolyte interface and the created electric field effectively dopes the graphene due to the extremely small separation between the graphene and the ion accumulation layer. Electrostatic doping of charge carriers in graphene shifts the Fermi level and this causes the inter-band transitions to be blocked by Pauli blocking. For Pauli blocking to take place, one should shift the Fermi level (E_F_) above the half of the incoming photon energy. This blocks the inter-band transitions and makes the graphene more transparent by blocking optical absorption mechanism of the material. By blocking the inter-band transitions, we achieved the light modulation over a broad range of wavelengths with the large area graphene supercapacitors. We achieve more efficient transmittance modulation in the case of smooth-Cu-graphene based supercapacitors due to the lower sheet resistance and high coverage of the smooth-Cu-graphene film. Figure 6(d) shows the transmittance versus voltage plot for both rough and smooth-Cu-graphene based supercapacitors at 700 nm. Transmittance modulation at single wavelength gives clear picture about the efficiency of using smooth-Cu-graphene in large area optoelectronic devices. Maximum modulation obtained from rough-Cu- graphene based supercapacitor is 1.16% at 4.4 V while the smooth-Cu-graphene based supercapacitor provides 2.35%. The transmittance spectra shown in Fig. 6(b,c) is a spectral distribution of the change of the transmission and it has a step-like behavior with transition centre at 2E_F_. Therefore we were able to extract the Fermi energy values corresponding to each curve. Figure 6(e) shows the Fermi energy level shift of the rough and smooth-Cu-graphene with respect to applied bias voltage. According to our observation, maximum Fermi level shift obtained by using rough-Cu-graphene is 0.89 eV while for the smooth-Cu-graphene it is 1.13 eV at 4.4 V. The small Fermi level difference between the rough-Cu-graphene and smooth-Cu-graphene (max. 6 meV) at lower voltages is due to the unintentional doping of graphene during the Cu etching and transfer processes.

Here the limiting factor for the bias voltage is the electrochemical window of the electrolyte used in the supercapacitor structure. Since our devices use mutual gating between top and bottom graphene electrodes, they are limited with the two times of the electrochemical window of the electrolyte (1-Butyl-3-methylimidazolium hexafluorophosphate) which is around 4.5 V. In mutual gating; the Dirac points of the top and bottom graphene shifts with respect to each other leading to two different dips in the capacitance modulation. Figure 6(f) shows the capacitance and resistance modulation of the graphene supercapacitor with respect to the applied bias voltage. As the voltage is increased to both polarizations, we observed a dramatic capacitance and resistance change due to the electrostatic doping of the top and bottom graphene layers. Detailed explanation of the physics behind this observations can be found in our previous work^29^.

As a result, we obtained a more efficient transmittance modulation up to two times (at 700 nm) by using smooth-Cu-graphene in supercapacitors operating as optical modulators. We also observed 27% more efficient Fermi level shift compared to rough-Cu-foils (at 4.4 V). By using electrolytes with broader electrochemical windows, the enhancement in transmittance and Fermi energy shift by using smooth-Cu-graphene can be further demonstrated in optoelectronic devices.

## Discussion

In summary, we demonstrated that using the commercially available ultra-smooth Cu foils (e.g. the one typically used in lithium-ion batteries) in CVD synthesis holds great promise for high performance in large area graphene based devices. Recent industrial progress of large scale production of smooth Cu foils, for application such as lithium-ion batteries, can also provide opportunities to improve the quality of graphene grown by CVD. After a detailed structural comparison between the rough and smooth-Cu-graphene, we studied the performance improvements in the transistors based on smooth-Cu-graphene by comparing them to transistors based on rough-Cu-graphene. To demonstrate the promises of the smooth-Cu-graphene on large area, we fabricated flexible graphene electrodes and mapped the transmittance by optical scan. These electrodes enabled us to fabricate graphene-based flexible supercapacitors operating as voltage controlled optical modulators. By enlarging the previously demonstrated supercapacitor structure, we examined the effects of usage of rough and smooth-Cu-graphene in the large area electro-optic devices. As against previous works relating the Cu surface morphology to resulting graphene quality, our method does not require chemical treatment or deposition of Cu foils. The effectiveness of our approach is two-fold:

1. The lower price of commercially available ultra-smooth Cu foils (1 $ per m^2^) that we used in this study makes the costs affordable for large scale production of graphene. Cu surface modification techniques also add to the overall cost to the graphene growth with the standard Cu foils which itself costs around 115 $ per m^2^.
2. Since there is a growing interest in realizing the electronics over large area and flexible substrates such as soft plastics and even paper^36^,^37^ synthesis of graphene in large areas with a high quality plays a crucial role in the applications. So far the flexible electronics landscape has been occupied by the low-mobility organic semiconductors^37^,^38^. But, recently major advances have also been reported through silicon^39^,^40^, metal oxides devices^41^, and graphene based flexible optoelectronics^42^ and sensing^43^. Our work will significantly advance these recent progresses.

We believe that large scale and low cost synthesis of high quality graphene films and the compatibility of our method to the roll-to-roll fabrication would open an avenue through the realization of graphene based flexible optoelectronic systems such as cell phones with roll-up displays, e-paper, radio-frequency identification (RFID) tags, as well as medical patches that can be attached to the skin to deliver drugs or monitor vital signs, and tactile or electronic skin for robotics and prosthetics^36^,^37^,^44^,^45^.

## Methods

### CVD Synthesis of Graphene

For single layer graphene, at 1035 °C methane was sent into growth chamber in company with hydrogen (Partial pressures of CH_4_ and H_2_ gases are 285 mTorr and 40 mTorr and corresponding rate of flows are 25 sccm and 4 sccm respectively). We annealed the Cu substrates for half an hour at 1035 °C before the growth. Hydrogen flow was kept constant during the heating, annealing, growth and cooling. After 10 minutes of growth, samples were left for fast cooling to room temperature. The growth took place in a 8 inch diameter quartz chamber. We used both the commercially available 20 μm thick smooth Cu foils (*Mitsui mining and smelting co., LTD, B1-SBS)* and the standard 25 μm *Alfa Aesar* Cu foil (*item* #*13382*) as growth substrates in the same CVD chamber.

### Transfer of Graphene

For the transfer of graphene layers to the rigid substrates we used photoresist (PR) drop casting method. We drop casted thick droplets of S1813 photoresist on graphene holding Cu surfaces and waited overnight at 70 °C to gently harden the PR. Then hardened PR on graphene holding Cu was put into the dense FeCl_3_ solution for fast etching of Cu foils. When the Cu foil was completely etched away, graphene holding PR was put into DI water surface for 30 minutes to remove the FeCl_3_ residues. After air drying, we placed the graphene holding hardened PR on the target substrate and applied 110 °C for 1 minute to reflow the PR. During reflow, PR liquefies and releases the graphene layers on target substrate. Removing PR with acetone yields large area graphene on quartz and SiO_2_/Si substrates.

We also used lamination technique to transfer large area graphene on PVC. At 100 °C PVC becomes active and exhibits conformal sticking to the graphene holding side of ultra-smooth Cu foils when laminated through the hot rollers. Etching the Cu foils in FeCl_3_ solution yields the graphene on flexible PVC substrates.

### Raman spectroscopy

WITec Alpha 300 M Raman microscope system with 50x microscope objectives was used for Raman mapping. The excitation wavelength was 532 nm.

### Fabrication of transistors and electrical characterization

In order to fabricate the graphene based FETs, first conventional UV-photolithographic techniques were used to shape the source and drain electrodes on graphene. Then, thermal evaporation technique was used for metallization of the electrodes. The source and drain electrodes are 50 nm gold on 5 nm titanium adhesive layer. After lift-off step, we isolated the graphene layer by using reactive ion etching so that the graphene layers is aligned between the source and drain electrodes to define the channels. Transfer and output characteristics of the graphene based transistors were performed with HP4142B semiconductor parameter analyser. We recorded the drain current as a function of gate voltage at constant drain voltage. We repeat the measurements for devices having various channel lengths.

### Optical transmittance measurements

The transmittance measurements were performed by using Bruker Vertex 70 V FTIR spectrometer integrated with silicon photodetector working in visible and near-IR. We worked in the wavelength range between 400 nm to 1100 nm. We biased the graphene based transistors using Keithley 2400 source measure unit during the transmittance measurements. The leakage current is also recorded during the measurement.

## Additional Information

**How to cite this article**: Polat, E. O. *et al.* Synthesis of Large Area Graphene for High Performance in Flexible Optoelectronic Devices. *Sci. Rep.* **5**, 16744; doi: 10.1038/srep16744 (2015).

## Supplementary Material

Supplementary Information
