# Supplementary material for: Synthesis of Large Area Graphene for High Performance in Flexible Optoelectronic Devices
Source: Sci Rep. 2015 Nov 18;5:16744. doi: 10.1038/srep16744 (PMC4649757; doi:10.1038/srep16744)
Supplement: Supplementary Information [file srep16744-s1.pdf]

Supplementary Information for

Synthesis of Large Area Graphene for High Performance in Flexible Optoelectronic Devices

Emre O. Polat[1], Osman Balci[2], Nurbek Kakenov[2], Hasan Burkay Uzlu[2], Coskun Kocabas[2†], Ravinder Dahiya[1†]

[1]Electronics and Nanoscale Engineering, University of Glasgow, Glasgow, G12 8QQ, UK

[2]Department of Physics, Bilkent University, 06800, Ankara, Turkey

† Corresponding authors

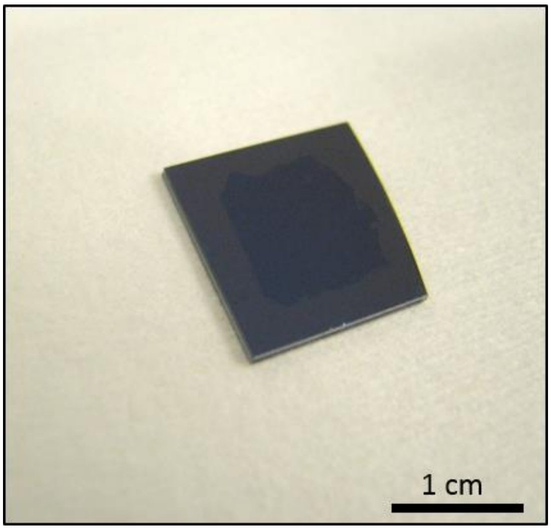

Figure S1: Photograph of smooth-Cu-graphene transferred to 100 nm SiO2 on Si.

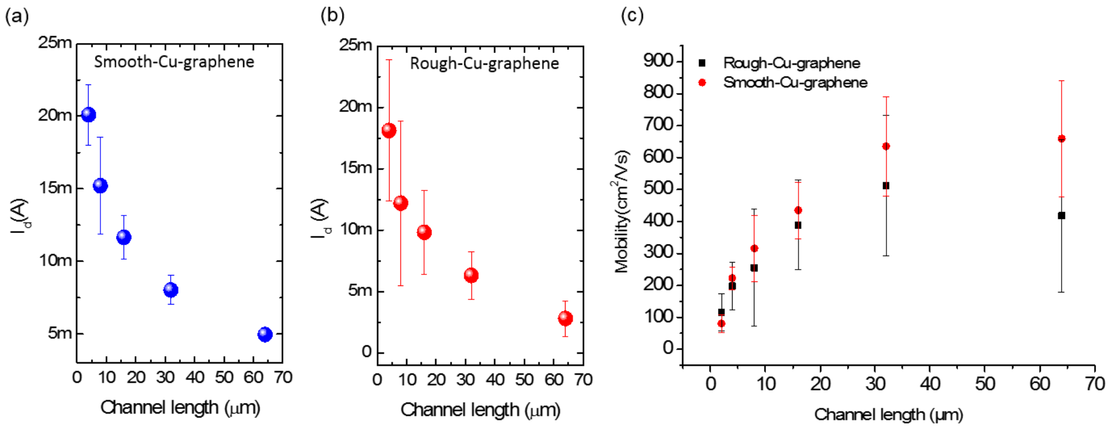

Figure S2: Device statistics of graphene based transistors. ***a,b,*** *On-current against channel length for the smooth and rough-Cu-graphene. The variation in the rough-Cu-graphene based transistors are higher and the on-current values are lower compared to smooth-Cu-graphene based transistors. Due to low integrity of rough-Cu-graphene, device variation is increasing with the channel length.* ***c,*** *Extracted field effect mobility values over 10 identical transistor with error bars.*

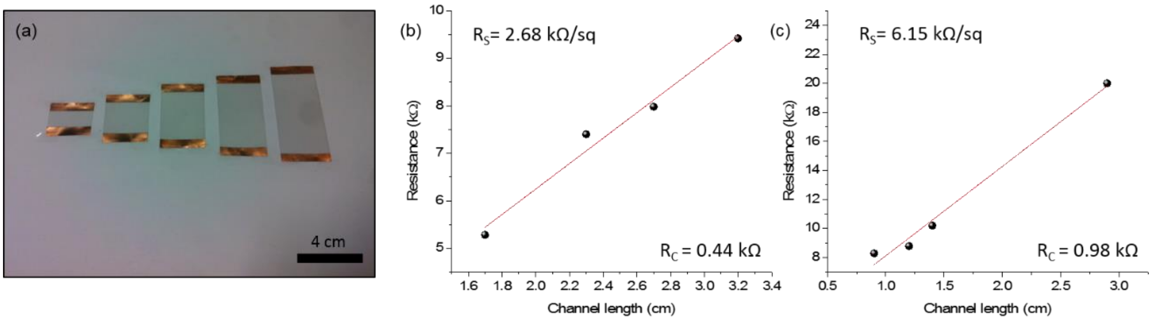

Figure S3: ***a,*** *Photograph of the transfer length measurement using large area graphene electrodes. We recorded the resistance values by changing the channel length and plotted with respect to channel length.* ***b,c,*** *Transfer line measurements for smooth-Cu-graphene and rough-Cu-graphene respectively. The slope yields the sheet resistance (R_S) and the intercept point gives the contact resistance (R_c) of the graphene electrodes. According to our TLM measurements the sheet resistance for smooth-Cu-graphene 2.68 kΩ/sq while rough-Cu-graphene has a sheet resistance of 6.15 kΩ/sq.*

.
